# A National Prediction Model for PM_2.5_ Component Exposures and Measurement Error–Corrected Health Effect Inference

**DOI:** 10.1289/ehp.1206010

**Published:** 2013-06-11

**Authors:** Silas Bergen, Lianne Sheppard, Paul D. Sampson, Sun-Young Kim, Mark Richards, Sverre Vedal, Joel D. Kaufman, Adam A. Szpiro

**Affiliations:** 1 Department of Biostatistics; 2 Department of Environmental and Occupational Health Sciences, and; 3 Department of Statistics, University of Washington, Seattle, Washington, USA

## Abstract

Background: Studies estimating health effects of long-term air pollution exposure often use a two-stage approach: building exposure models to assign individual-level exposures, which are then used in regression analyses. This requires accurate exposure modeling and careful treatment of exposure measurement error.

Objective: To illustrate the importance of accounting for exposure model characteristics in two-stage air pollution studies, we considered a case study based on data from the Multi-Ethnic Study of Atherosclerosis (MESA).

Methods: We built national spatial exposure models that used partial least squares and universal kriging to estimate annual average concentrations of four PM_2.5_ components: elemental carbon (EC), organic carbon (OC), silicon (Si), and sulfur (S). We predicted PM_2.5_ component exposures for the MESA cohort and estimated cross-sectional associations with carotid intima-media thickness (CIMT), adjusting for subject-specific covariates. We corrected for measurement error using recently developed methods that account for the spatial structure of predicted exposures.

Results: Our models performed well, with cross-validated *R*^2^ values ranging from 0.62 to 0.95. Naïve analyses that did not account for measurement error indicated statistically significant associations between CIMT and exposure to OC, Si, and S. EC and OC exhibited little spatial correlation, and the corrected inference was unchanged from the naïve analysis. The Si and S exposure surfaces displayed notable spatial correlation, resulting in corrected confidence intervals (CIs) that were 50% wider than the naïve CIs, but that were still statistically significant.

Conclusion: The impact of correcting for measurement error on health effect inference is concordant with the degree of spatial correlation in the exposure surfaces. Exposure model characteristics must be considered when performing two-stage air pollution epidemiologic analyses because naïve health effect inference may be inappropriate.

Citation: Bergen S, Sheppard L, Sampson PD, Kim SY, Richards M, Vedal S, Kaufman JD, Szpiro AA. 2013. A national prediction model for PM_2.5_ component exposures and measurement error–corrected health effect inference. Environ Health Perspect 121:1017–1025; http://dx.doi.org/10.1289/ehp.1206010

## Introduction

The relationship between air pollution and adverse health outcomes has been well documented (Pope et al. 2002; Samet et al. 2000). Many studies focus on particulate matter, specifically particulate matter ≤ 2.5 μm in aerodynamic diameter (PM_2.5_) (Kim et al. 2009; Miller et al. 2007). Health effects of PM_2.5_ may depend on characteristics of the particles, including shape, solubility, pH, or chemical composition (Vedal et al., in press), and a deeper understanding of these differential effects could help inform policy. One of the challenges in assessing the impact of different chemical components of PM_2.5_ in an epidemiologic study is the need to assign exposures to study participants based on monitoring data from different locations (i.e., spatially misaligned data). When doing this for many components, the prediction procedure needs to be streamlined in order to be practical. Whatever the prediction algorithm, using the estimated rather than true exposures induces measurement error in the subsequent epidemiologic analysis. Here we describe a flexible and efficient prediction model that can be applied on a national scale to estimate long-term exposure levels for multiple pollutants and that implements existing methods of correcting for measurement error in the health model.

Current methods for assigning exposures include land-use regression (LUR) with geographic information system (GIS) covariates (Hoek et al. 2008) and universal kriging, which also exploits residual spatial structure (Kim et al. 2009; Mercer et al. 2011). Often hundreds of candidate correlated GIS covariates are available, necessitating a dimension reduction procedure. Variable selection methods that have been considered in the literature include exhaustive search, stepwise selection, and shrinkage by the “lasso” (Mercer et al. 2011; Tibshirani 1996). However, variable selection methods tend to be computationally intensive, feasible perhaps when considering a single pollutant but quickly becoming impractical when developing predictions for multiple pollutants. A more streamlined alternative is partial least squares (PLS) regression (Sampson et al. 2009), which finds a small number of linear combinations of the GIS covariates that most efficiently account for variability in the measured concentrations. These linear combinations reduce the covariate space to a much smaller dimension and can then be used as the mean structure in a LUR or universal kriging model in place of individual GIS covariates. This provides the advantages of using all available GIS covariates and eliminating potentially time-consuming variable selection processes.

Using exposures predicted from spatially misaligned data rather than true exposures in health models introduces measurement error that may have implications for ^^^^^β_x_, the estimated health model coefficient of interest (Szpiro et al. 2011b). Berkson-like error that arises from smoothing the true exposure surface may inflate the SE of ^^^^^β_x_. Classical-like error results from estimating the prediction model parameters and may bias ^^^^^β_x_ in addition to inflating its SE. Bootstrap methods to adjust for the effects of measurement error have been discussed by Szpiro et al. (2011b).

Here we present a case study to illustrate a holistic approach to two-stage air pollution epidemiologic modeling, which includes exposure modeling in the first stage and health modeling that incorporates measurement error correction in the second stage. We build national exposure models using PLS and universal kriging, and employ them to estimate long-term average concentrations of four chemical species of PM_2.5_—elemental carbon (EC), organic carbon (OC), silicon (Si), and sulfur (S)—selected to reflect a variety of different PM_2.5_ sources and formation processes (Vedal et al., in press). After developing the exposure models, we derive predictions for the Multi-Ethnic Study of Atherosclerosis (MESA) cohort. These predictions are used as the covariates of interest in health analyses assessing associations between carotid intima-media thickness (CIMT), a subclinical measure of atherosclerosis, and exposure to PM_2.5_ components. We apply measurement error correction methods to account for the fact that predicted rather than true exposures are being used in these health models. We discuss our results and their implications with regard to the effect of spatial correlation in exposure surfaces on estimated associations between exposures and health outcomes.

## Data

**Figure 1:**
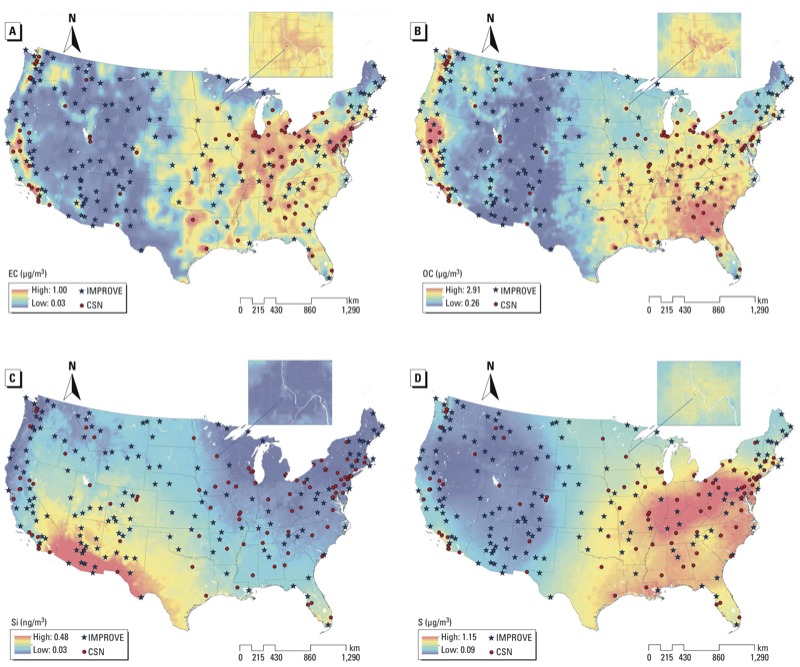
Locations of IMPROVE and CSN monitors and predicted national average PM_2.5_ component concentrations from final predictions models. (*A*) EC, (*B*) OC, (*C*) Si, and (*D*) S. Insets show predictions for St. Paul, MN.

*Monitoring data*. Data on EC, OC, Si, and S were collected to build the national models. These data consisted of annual averages from 2009–2010 as measured by the Interagency Monitoring for Protected Visual Environments (IMPROVE) and Chemical Speciation Network (CSN) of the U.S. Environmental Protection Agency (U.S. EPA 2009). The IMPROVE monitors are a nationwide network located mostly in remote areas. The CSN monitors are located in more urban areas. These two networks provide data that are evenly dispersed throughout the lower 48 states (Figure 1).

All IMPROVE and CSN monitors that had at least 10 data points per quarter and a maximum of 45 days between measurements were included in our analyses. Si and S measurements were averaged over 1 January 2009–31 December 2009. The EC/OC data set consisted of measurements from 204 IMPROVE and CSN monitors averaged over 1 January 2009–31 December 2009, and measurements from 51 CSN monitors averaged over 1 May 2009–30 April 2010. We used the latter period because the measurement protocol used by CSN monitors prior to 1 May 2009 was incompatible with the IMPROVE network protocol. Comparing values averaged over 1 May 2009–30 April 2010 to those averaged over 1 January 2009–31 December 2009 indicated little difference between the time periods (data not shown). The annual averages were square-root transformed prior to modeling.

*Geographic covariates*. Approximately 600 LUR covariates were available for all monitor and subject locations. These included distances to A1, A2, and A3 roads [census feature class codes (CFCCs; U.S. Census Bureau 2013)]; land use within a given buffer; population density within a given buffer; and Normalized Difference Vegetation Index (NDVI; National Oceanic and Atmospheric Administration 2013), which measures the level of vegetation in a monitor’s vicinity. CFCC A1 roads are limited-access highways; A2 and A3 roads are other major roads such as county and state highways without limited access (Mercer et al. 2011). For NDVI a series of 23 monitor-specific, 16-day composite satellite images were obtained, and the pixels within a given buffer were averaged for each image. PLS incorporated the 25th, 50th, and 75th percentile of these 23 averages. The median of “high-vegetation season” image averages (defined as 1 April–30 September) and “low-vegetation season” averages (1 October–31 March) were also included. The geographic covariates were pre-processed to eliminate LUR covariates that were too homogeneous or outlier-prone to be of use. Specifically, we eliminated variables with > 85% identical values, and those with the most extreme standardized outlier > 7. We log-transformed and truncated all distance variables at 10 km, and computed additional “compiled” distance variables such as minimum distance to major roads and distance to any port. These compiled variables were then subject to the same inclusion criteria. All selected covariates were mean-centered and scaled by their respective SDs.

*MESA cohort.* MESA is a population-based study that began in 2000, with a cohort consisting of 6,814 participants from six U.S. cities: Los Angeles, California; St. Paul, Minnesota; Chicago, Illinois; Winston-Salem, North Carolina; New York, New York; and Baltimore, Maryland. Four ethnic/racial groups were targeted: white, Chinese American, African American, and Hispanic. All participants were free of physician-diagnosed cardiovascular disease at time of entrance. [For additional details about the MESA study, see Bild et al. (2002).] These participants were also utilized in the Multi-Ethnic Study of Atherosclerosis and Air Pollution (MESA Air), an ancillary study to MESA funded by the U.S. EPA to study the relationship between chronic exposure to air pollution and progression of subclinical cardiovascular disease (Kaufman et al. 2012). Both the MESA and MESA Air studies were approved by the institutional review board (IRB) at each site, including the IRBs at the University of California, Los Angeles (Los Angeles, CA), Columbia University (New York, NY), Johns Hopkins University (Baltimore, MD), the University of Minnesota (Minneapolis-St. Paul, MN), Wake Forest University (Winston-Salem, NC), and Northwestern University (Evanston, IL). All subjects gave written informed consent.

We selected the CIMT end point in MESA as the health outcome for our case study. CIMT, a subclinical measure of atherosclerosis, was measured by B-mode ultrasound using a GE Logiq scanner (GE Healthcare, Wauwatosa, WI), and the end point was quantified as the right far wall CIMT measures conducted during MESA exam 1, which took place during 2000–2002 (Vedal et al., in press). We considered the 5,501 MESA participants who had CIMT measures during exam 1; our analysis was based on the 5,298 MESA participants who had CIMT measures during exam 1 and complete data for all selected model covariates.

## Methods

The first stage of the two-stage approach included building the exposure models using PLS as the covariates in universal kriging models. We used cross-validation (CV) to select the number of PLS scores, determine how reliable predictions from each exposure model were, and assess the extent to which spatial structure was present for each pollutant. The health modeling stage of the two-stage approach included the health models we fit and the measurement error correction methods we employed. [For more detailed technical exposition, see Bergen et al. (2012).]

*Spatial prediction models.* Notation. Let X_t_* denote the N* × 1 vector of observed square-root transformed concentrations at monitor locations; R* the N* × *p* matrix of geographic covariates at monitor locations; X*_t_* the *N* × 1 vector of unknown square-root transformed concentrations at the unobserved subject locations; and R the *N* × *p* matrix of geographic covariates at the subject locations. Note that for our exposure models, X_t_* and X*_t_* are dependent variables, and R* and R are independent variables. We used PLS to decompose R* into a set of linear combinations of much smaller dimension than R*. Specifically,

R*H = T*.

Here, H is a *p* × *k* matrix of weights for the geographic covariates, and T* is an N* × *k* matrix of PLS components or scores. These scores are linear combinations of the geographic covariates found in such a way that they maximize the covariance between X_t_* and all possible linear combinations of R*. One might notice similarities between PLS and principal components analysis (PCA). Although the two methods are similar in that they are both dimension reduction methods, the scores from PLS maximize the covariance between X_t_* and all other possible linear combinations of R*, whereas the scores from PCA are chosen to explain as much as possible the covariance of R*. [For more details see Sampson et al. (2013)]. PLS scores at unobserved locations are then derived as T = RH.

Once the PLS scores T and T* were obtained for the subject and monitoring locations, respectively, we assumed the following joint model for unobserved and observed exposures:


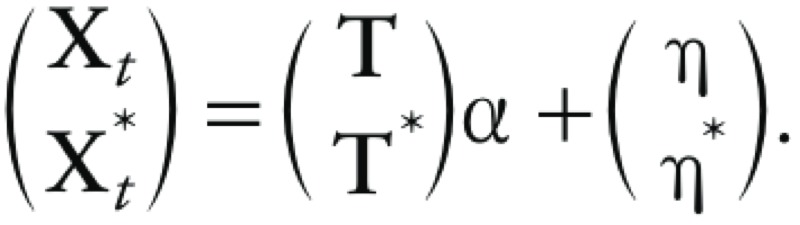
[1]

Here α is a vector of regression coefficients for the PLS scores, and η and η* are *N* × 1 and N* × 1 vectors of errors, respectively. Our primary exposure models assumed that the error terms exhibited spatial correlation that could be modeled with a kriging variogram parameterized by a vector of parameters θ⊇= (τ^2^, σ^2^, ϕ) (Cressie 1992). The nugget, τ^2^, is interpretable as the amount of variability in the pollution exposures that is not explained by spatial structure; the partial sill, σ^2^, is interpretable as the amount of variability that is explained by spatial structure; and the range, ϕ, is interpretable as the maximum distance between two locations beyond which they may no longer be considered spatially correlated. We estimated these parameters and the regression coefficients α via profile maximum likelihood. Once these parameters were estimated, we obtained predictions at unobserved locations by taking the mean of X*_t_* conditional on X_t_* and the estimated exposure model parameters. Because our measurement error correction methods rely on a correctly specified exposure model, we took care to choose the best-fitting kriging variogram to model our data. We initially fit exponential variograms for all four pollutants and investigated whether plots of the estimated variogram appeared to fit the empirical variogram well. If they appeared to fit poorly, we investigated spherical and cubic variograms. The exponential variogram fit well for EC, OC, and S, but provided a poor fit for Si (data not shown). We therefore examined cubic and spherical variograms and found the spherical variogram provided a much better fit and used it to model Si in our exposure models.

As a comparison to our primary kriging models, we also derived predictions from PLS alone without fitting a kriging variogram. This is analogous to a pure LUR model but using the PLS scores instead of actual geographic covariates. For this analysis η and η* were assumed to be independent, and α was estimated using a least-squares fit to regression of X_t_* on T*. PLS-only predictions at the unobserved locations were then derived as the fitted values from this regression using the PLS scores at the subject locations.

CV and model selection. We used 10-fold CV (Hastie et al. 2001) to assess the models’ prediction accuracy, to select the number of PLS components to use in the final prediction models, and to compare predictions generated using PLS only to our primary models, which used both PLS and universal kriging. Data were randomly assigned to 1 of 10 groups. One group (a “test set”) was omitted, and the remaining groups (a “training set”) were used to fit the model and generate test set predictions. Each group played the role of test set until predictions were obtained for the entire data set. At each iteration, the following steps were taken to cross-validate our primary models (similar steps were followed to derive cross-validated predictions that used PLS only):

PLS was fit using the training set, and *K* scores were computed for the test set, for *K* = 1,...,10.
Universal kriging parameters θ and coefficients α were estimated via profile maximum likelihood using the training set. The first *K* PLS scores correspond to T* in Equation 1, for *K* = 1,...,10.
Predictions were derived using the first *K* PLS components and the corresponding universal kriging, using kriging parameters estimated from the training set.

We used the R package pls to fit the PLS. universal kriging was performed using the R package geoR. The best-performing models were selected out of those that used both PLS and kriging based on their cross-validated root mean squared error of prediction (RMSEP) and corresponding *R*^2^. For a data set with N* observations and corresponding predictions, the formulae for these performance metrics are given by


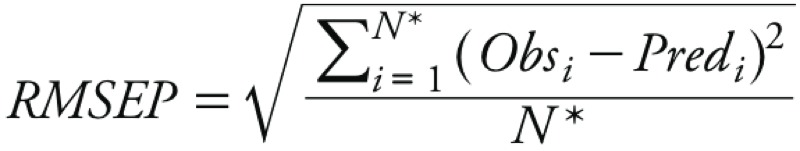
[2]

and


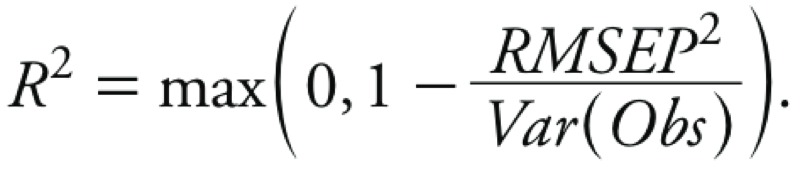
[3]

These metrics are sensitive to scale; accordingly, they are useful for evaluating model performance for a given pollutant but not for comparing models across pollutants.

*Health modeling*. Disease model. Multivariable linear regression models were used to estimate the effects of each individual PM_2.5_ component exposure on CIMT. Each model included a single PM_2.5_ component along with a vector of subject-specific covariates. Let Y be the 5,298 × 1 vector of health outcomes for the 5,298 MESA participants included in the analysis, W the 5,298 × 1 vector of exposure predictions on the untransformed scale, and Z a matrix of potential confounders. We assumed linear relationships between Y, the true exposures, and Z, and fit the following equation via ordinary least squares (OLS):

*E*(*Y*) = β_0_ + *W*β*_x_ + Z*β*_z_.* [4]

Measurement error correction. The model in Equation 4 was fit using the predicted exposures W instead of the true exposures as the covariate of interest. Using predictions rather than true exposures in health modeling introduces two sources of measurement error that potentially influence the behavior of ^^^^^β_x_. Berkson-like error arises from smoothing the true exposure surface and could inflate the SE of ^^^^^β_x_. Classical-like error arises from estimating the exposure model parameters α and θ. The classical-like error potentially inflates the SE of ^^^^^β_x_ and could also bias the point estimate. We implemented the parameter bootstrap, an efficient method to assess and correct for the effects of measurement error. [See Szpiro et al. (2011b) for additional background and details.]

We used the parameter bootstrap in the context of predictions that use both PLS and universal kriging; the approach would be very similar if PLS alone was used (although we did not implement that correction here).

Estimate a sampling density for ^^^^^α and ^^^^^θ with a multivariate normal distribution.
For *j* = 1,...,*B* bootstrap samples
Simulate new “observed” bootstrap exposures at monitoring locations from Equation 1 and health outcomes from Equation 4.
Sample new exposure model parameters and, from the sampling density estimated in step 1, using a constant covariance matrix multiplied by a scalar λ ≥ 0. λ controls the variability of (^^^^^α*_j_,* ^^^^^θ*_j_*): the larger λ is, the greater the variability of (^^^^^α*_j_,* ^^^^^θ*_j_*).
Use the simulated health outcomes and newly-sampled exposure model parameters to derive W*_j_*.
Calculate ^^^^^β_x,j_ using W*_j_* by OLS.
  1. Let E_λ_(^^^^^β_x_^B^) denote the empirical mean of the ^^^^^β_x,j_. The estimated bias is defined as Bias_λ_(^^^^^β_x_) = E_λ_(^^^^^β_x_^B^)–E_0_(^^^^^β_x_^B^) with corresponding bias-corrected effect estimate β_x,λ_^corrected^ = ^^^^^β_x_–Bias_λ_(^^^^^β_x_).
  2. Estimate the bootstrap SE as

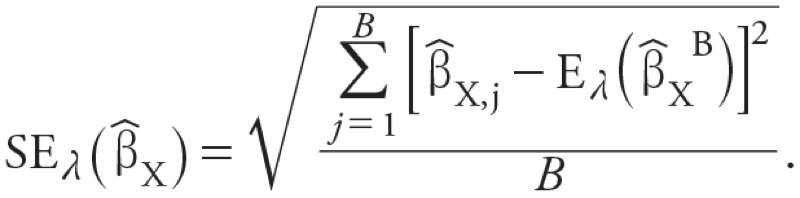
[5]

For our implementation of the parameter bootstrap, we set *B* = 30,000 and λ⊇= 1.

The goal of the parameter bootstrap is to approximate the sampling properties of the measurement error-impacted ^^^^^β_x_ that would be estimated if we performed our two-stage analysis with many actual realizations of monitoring observations and subject health data sets. Accordingly, step 2(a) gives us *B* new “realizations” of our data. For λ⊇= 1, step 2(b) accounts for the classical-like error by resampling the exposure model parameters. Step 2(c) accounts for the Berkson-like error by smoothing the true exposure surface. Step 2(d) then calculates *B* new ^^^^^β_x,j_’s, the sampling properties of which have incorporated all sources of measurement error. Comparing these to the mean of bootstrapped ^^^^^β_x,j_ derived using fixed exposure model parameters (i.e., λ⊇= 0) gives us an approximation of the bias induced by the classical-like error (step 3), and the empirical SD approximates the SE that accounts for both sources of measurement error (step 4).

We also implemented the parameter bootstrap for λ = 0. This is equivalent to the “partial parametric bootstrap” described by Szpiro et al. (2011b), which accounts for the Berkson-like error only because the exposure surface is still smoothed, but with fixed parameters.

A desirable trait of the parameter bootstrap is the ability to “tune” the amount of the classical-like error by varying λ, which allows us to investigate how variability in the sampling distribution of (^^^^^α*_j_,* ^^^^^θ*_j_*) affects the bias of ^^^^^β_x_. This can be useful in refining our bootstrap bias estimates by simulation extrapolation (SIMEX) (Stefanski and Cook 1995). (For additional information on our approach to SIMEX and the results of applying it to the MESA data, see Supplemental Material, pp. 2–3 and Figure S1.)

## Results

*Data*. Monitoring data. Mean concentrations of the four pollutants according to monitoring network are shown in Table 1. EC and OC concentrations measured by CSN monitors tended to be higher than concentrations measured by IMPROVE monitors. Average Si and S concentrations measured by CSN monitors were also higher than the IMPROVE averages; however, relative to their SDs, the differences between CSN and IMPROVE monitors in Si and S concentrations were not as great as the differences between EC and OC concentrations.

**Table 1:** Summary data for observed pollution concentrations (mean ± SD) at monitoring networks; predicted concentrations (mean ± SD) for the MESA cohort at exam 1 and summaries of selected LUR covariates.

Covariates	IMPROVE	CSN	All monitors	MESA Air
Sites (*n*)	190	98	288	5501
EC (μg/m^3^)	0.19±0.18	0.66±0.24	0.37±0.30	0.74±0.18
OC (μg/m^3^)	0.93±0.55	2.23±0.71	1.43±0.88	2.17±0.36
Si (ng/m^3^)	0.16±0.12	0.10±0.09	0.14±0.11	0.09±0.03
S (μg/m^3^)	0.41±0.27	0.69±0.25	0.51±0.29	0.78±0.15
Sites <150m to an A1 road[*n* (%)]	4 (2)	3 (3)	7 (2)	249 (6)
Sites <150m to an A3 road[*n* (%)]	36 (19)	43 (44)	79 (27)	2,763 (50)
Median distance to comm (m)	4,696	127	1,235	302
Median pop dens^*a*^ (persons/mi^2^)	3	805	20	3,496
NDVI^*b*^	150	140	146	137
Abbreviations: comm, commercial or service centers; pop dens, population density.^***a***^Persons per square mile for census block/block group to which monitor/­subject belongs. ^***b***^Median value of summer NDVI medians within 250-m buffer.				

Geographic covariates. The geographic variables that we used are listed in Table 2. Most of these variables were used for modeling all four pollutants, but not all. The following variables were used for modeling Si and S but not EC and OC: PM_2.5_ and PM_10_ emissions, streams and canals within a 3-km buffer, other urban or built-up land use within a 400-m buffer, lakes within a 10-km buffer, industrial and commercial complexes within a 15-km buffer, and herbaceous rangeland within a 3-km buffer. On the other hand, the following variables were used for modeling EC and OC but not Si and S: industrial land use within 1- and 1.5-km buffers.

**Table 2:** LUR covariates (Figure 2 abbreviations) and (where applicable) covariate buffer sizes that made it through preprocessing and were considered by PLS.

Abbreviation	Variable description	Buffer sizes
Distance to features	A1 road^*a*^	NA
	Nearest road^*a*^	NA
	Airport^*a*^	NA
	Large airport^*a*^	NA
	Port^*a*^	NA
	Coastline^*a,b*^	NA
	Commercial or service center^*a*^	NA
	Railroad^*a*^	NA
	Rail yard^*a*^	NA
SO_2_	SO_2_ Emissions^*c*^	30km
PM_2.5_	PM_2.5_^*c,d*^	30km
PM_10_	PM_10_^*c,d*^	30km
NO_x_	NO_x_^*c*^	30km
Population	Population density	500m, 1km, 1.5km, 2km, 2.5km, 3km, 5km, 10km, 15km
NDVI–winter	Median winter	250m, 500m, 1km, 2.5km, 5km, 7.5km, 10km
NDVI–summer	Median summer	250m, 500m, 1km, 2.5km, 5km, 7.5km, 10km
NDVI–Q75	75th percentile	250m, 500m, 1km, 2.5km, 5km, 7.5km, 10km
NDVI–Q50	50th percentile	250m, 500m, 1km, 2.5km, 5km, 7.5km, 10km
NDVI–Q25	25th percentile	250m, 500m, 1km, 2.5km, 5km, 7.5km, 10km
Transport	Transportation, communities, and utilities	750m, 3km, 5km, 10km, 15km
Transition	Transitional areas	15km
Stream	Streams and canals	3km^*d*^, 5km, 10km, 15km
Shrub	Shrub and brush rangeland	1.5km, 3km, 5km, 10km, 15km
Residential	Residential	400m, 500m, 750m, 1km, 1.5km, 3km, 5km, 10km, 15km
Other urban	Other urban or built-up	400m^*d*^, 500m, 1.5km, 3km, 5km, 10km, 15km
Mixed range	Mixed rangeland	3km, 5km, 10km, 15km
Mixed forest	Mixed forest land	750m, 1km, 1.5km, 3km, 5km, 10km, 15km
Lakes	Lakes^*d*^	10 km
Industrial	Industrial	1km^*e*^, 1.5km^*e*^, 3km, 5km, 10km, 15km
Indust/comm	Industrial and commercial complexes^*d*^	15km
Herb range	Herbaceous rangeland	3km^*d*^, 5km, 10km
Green	Evergreen forest land	400m, 500m, 750m, 1km, 1.5km, 3km, 5km, 10km, 15km
Forest	Deciduous forest land	750m, 1km, 1.5km, 3km, 5km, 10km, 15km
Crop	Cropland and pasture	400m, 500m, 750m, 1km, 1.5km, 3km, 5km, 10km, 15km
Comm	Commercial and services	500m, 750m, 1km, 1.5km, 3km, 5km, 10km, 15km
A23	Total distance of A2 and A3 roads within buffer	100m, 150m, 300m, 400m, 500m, 750m, 1km, 1.5km, 3km, 5km
A1	Total distance of A1 roads within buffer	1km, 1.5km, 3km, 5km
Most variables were used in each of the four PM_2.5_ component models; however, the pre-processing procedure selected some variables for EC and OC that were not selected for Si and S, and vice versa because EC and OC monitoring locations were not identical to Si and S locations.^***a***^Truncated at 25km and log_10_ transformed. ^***b***^log_10_ and untransformed values both included. ^***c***^Tons per year of emissions from tall stacks. ^***d***^Variable used for modeling Si, S only. ^***e***^Variable used for modeling EC and OC only.		

The distributions of selected geographic covariates are shown according to monitoring network and MESA locations in Table 1. Although relatively few monitors belonging to either IMPROVE or CSN were within 150 m of an A1 road, there was a larger proportion of CSN monitors within 150 m of an A3 road (44%) than IMPROVE monitors (19%), consistent with the placement of CSN monitors in more urban locations compared with IMPROVE monitors (Table 1). The median distance to commercial and service centers was much smaller for CSN monitors (127 m vs. 4,696 m), and the median population density was much larger for CSN monitors (805 persons/mi^2^) than for IMPROVE monitors (only 3 persons/mi^2^). Median summer NDVI values within 250 m were slightly smaller for CSN monitors than for IMPROVE monitors, consistent with the placement of IMPROVE monitors in greener areas. Geographic covariate distributions among MESA participant locations were more consistent with the CSN monitors, as is especially evident for the number of sites < 150 m from an A3 road and median population density (Table 1). Density plots of the geographic covariates for monitoring and subject locations indicated noticeable overlap for all geographic covariates (data not shown), suggesting differences in geographic covariates between monitor and MESA locations were consistent with the concentration of MESA subjects in urban locations, not extrapolation beyond our data.

MESA cohort. Distributions of health model covariates among MESA cohort participants are summarized in Table 3. The mean CIMT (0.68 ± 0.19 mm); mean age (62 ± 10 years); sex (52% female); race (39% white, 12% Chinese American, 27% African American, and 22% Hispanic); and status (44% hypertension status and 15% statin use) were determined by questionnaire (Bild et al. 2002). The highest percentage of participants resided in Los Angeles (19.7%), but the distribution across the six cities was quite homogeneous. Only the 5,298 participants with complete data for all the selected model covariates listed in Table 3 were included in the analysis.

**Table 3:** Subject-specific covariates for the MESA cohort used in health modeling.

Variable	*n*	Mean±SD or %
CIMT	5,501	0.68±0.19
Age (years)	5,501	61.9±10.1
Weight (lb)	5,501	173.0±37.5
Height (cm)	5,501	166.6±10.0
Waist (cm)	5,500	97.8±14.1
Body surface area (m^2^)	5,501	1.9±0.2
BMI (kg/m^2^)	5,501	28.2±5.3
DBP	5,499	71.8±10.3
Sex
Female	2,872	52.2
Male	2,629	47.8
Race
White (Caucasian)	2,168	39.4
Chinese American	675	12.3
Black (African American)	1,459	26.5
Hispanic	1,199	21.8
Site
Winston-Salem	878	16.0
New York	867	15.8
Baltimore	776	14.1
St. Paul and Minneapolis	899	16.3
Chicago	998	18.1
Los Angeles	1,083	19.7
Education
Incomplete high school	916	16.7
Completed high school	991	18.0
Some college	1,571	28.6
Completed college	2,010	36.5
Missing	13	0.2
Income per year
<$12,000	566	10.3
$12,000–24,999	1,022	18.6
$25,000–49,999	1,543	28
$50,000–74,999	901	16.4
>$75,000	1,271	23.1
Missing	198	3.6
Hypertension
No	3,106	56.5
Yes	2,395	43.5
Statin use
No	4,681	85.1
Yes	817	14.9
Missing	3	0.1

*Spatial prediction models.* Model evaluation. The selected models corresponding to lowest cross-validated *R*^2^ all used PLS and universal kriging. For all four PM_2.5_ components and for all numbers of PLS scores, kriging improved prediction accuracy, as indicated by the *R*^2^ and RMSEP statistics for the selected prediction models corresponding to the best performing PLS-only and PLS + universal kriging models (Table 4). Comparing the *R*^2^ with and without universal kriging indicates that EC and OC were not much improved by kriging, whereas universal kriging improved prediction accuracy for Si and even more so for S. The ratio of the nugget to the sill (i.e., τ^2^/σ^2^) also supports improved predictions with spatial smoothing by kriging. For a fixed range, smaller values of this ratio indicate that concentrations at nearby locations receive greater weight when kriging. We see this relationship in Table 4 where τ^2^/σ^2^ was large when universal kriging did little to improve prediction accuracy, and very small when universal kriging helped improve prediction accuracy.

**Table 4:** Cross-validated R^2^ and RMSEP for each component of PM_2.5_, for both primary models and comparison PLS-only models, and the estimated kriging parame­ters from the likelihood fit on the entire data set for each pollutant.

Correction	Model	EC	OC	Si	S
		3 PLS scores	2 PLS scores	2 PLS scores	2 PLS scores
*R*^2^	PLS only	0.79	0.60	0.36	0.63
	PLS+UK	0.82	0.69	0.62	0.95
RMSEP	PLS only	0.11	0.22	0.10	0.13
	PLS+UK	0.10	0.20	0.08	0.05
Estimated UK parameters	(τ^2^)^*a*^	0.0074	0.0251	0.0043	0.0007
	(σ^2^)^*b*^	0.0025	0.0199	0.0086	0.0251
	(φ)^*c*^	413	304	2,789	2,145
	(τ^2^/σ^2^)	2.96	1.26	0.5	0.03
UK, universal kriging.^***a***^Nugget used in kriging. ^***b***^Partial sill used in kriging. ^***c***^Range used in kriging.					

As a sensitivity analysis we also carried out CV using nearest-monitor exposure estimates. This method performed very poorly for EC and OC (*R*^2^s of 0 and 0.06, respectively), relatively poorly for Si (*R*^2^ = 0.36), but performed well for S (*R*^2^ = 0.88).

**Figure 2:**
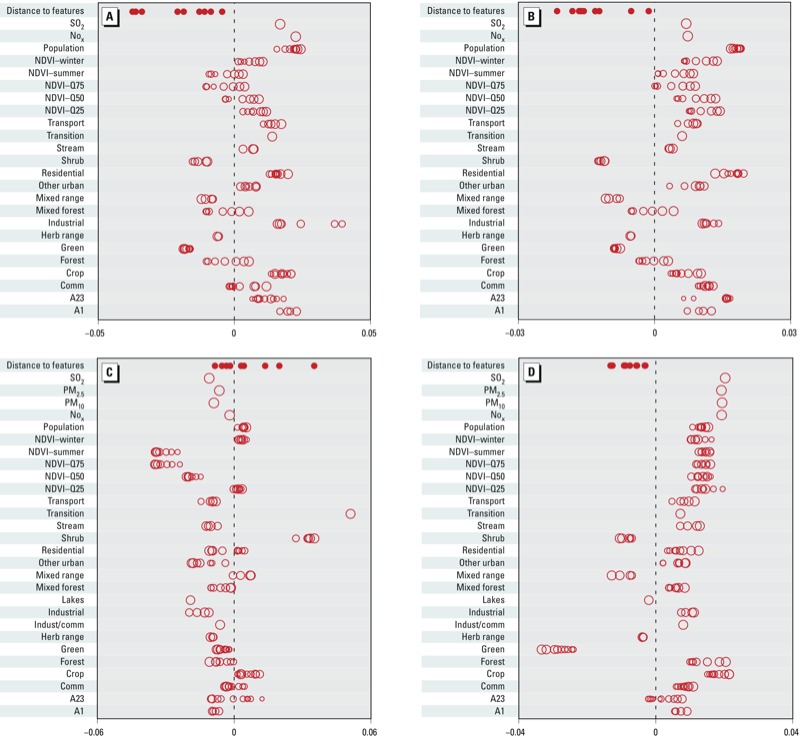
Coefficients of the PLS fit, where the coefficients describe the associations of each geographic covariate with exposure for (*A*) EC, (*B*) OC, (*C*) Si, and (*D*) S. The size of each circle represents covariate buffer size, with larger circles indicating larger buffers. Each closed circle for “distance to feature” represents a different feature (listed in Table 2): A1 road, nearest road, airport, large airport, port, coastline, commercial or service center, railroad, and rail yard. Variable abbreviations and buffer sizes are indicated in Table 2. Most of the variables shown here were used for modeling all four pollutants, but not all. Variables used for modeling Si and S but not EC and OC were PM2.5 and PM10 emissions, streams and canals within a 3-km buffer, other urban or built-up land use within a 400-m buffer, lakes within a 10-km buffer, industrial and commercial complexes within a 15-km buffer, and herbaceous rangeland within a 3-km buffer. The variables used for modeling EC and OC but not Si and S were industrial land use within 1- and 1.5-km buffers.

*Interpretation of PLS.* Figure 2 illustrates the geographic covariates that were most important for explaining pollutant variability. Specifically, Figure 2 summarizes the *p* × 1 vector m, the vector such that Rm equals the 5,298 exposures predicted with PLS only. Each element of m is a weight for a corresponding geographic covariate. Positive elements in m (i.e., values > 0 in Figure 2) indicate that higher values of the geographic covariate were associated with higher predicted exposure; the larger the absolute value of an element in m, the more the corresponding geographic covariate contributed to exposure prediction.

Population density was associated with larger predicted values of all pollutants, particularly for EC, OC, and S. Industrial land use within the smallest buffer was very predictive of EC and OC, and evergreen forest land within a given buffer was strongly predictive of decreases in S. NDVI, industrial land use, emissions, and line-length variables were positively associated with all exposures except Si, whereas all the distance-to-features variables were negatively associated with all exposures except Si. The NDVI variables were more important for prediction of OC and S than they were for EC. For Si, the NDVI and transitional land use variables appeared to be the most informative for prediction, with NDVI negatively and transitional land use positively associated with Si exposure. Distance to features appeared to be informative for all four pollutants.

Exposure predictions. Figure 1 shows predicted concentrations across the United States, with finer detail illustrated for St. Paul, Minnesota. The EC and OC predictions were much higher in the middle of urban areas, and quickly dissipated further from urban centers. S predictions were high across the midwestern and eastern states and in the Los Angeles area, and lower in the plains and mountains. Si predictions were low in most urban areas, and high in desert states.

Mean predicted EC and OC exposure concentrations predicted for MESA participants were 0.74 ± 0.18 and 2.17 ± 0.36 μg/m^3^, respectively (Table 1). Mean predicted Si and S exposure concentrations were 0.09 ± 0.03 ng/m^3^ and 0.78 ± 0.15 μg/m^3^, respectively.

*Health models.* The results from the naïve health model that did not include any measurement error correction, as well as the results from the health model that included bootstrap-corrected point estimates and SEs of ^^^^^β_x_, are displayed in Table 5. The naïve analysis indicated significant positive associations (*p* < 0.05) of CIMT with OC, Si, and S. There was also a positive but nonsignificant association between CIMT and EC. SEs for the EC and OC health effects were virtually unchanged when measurement error correction was implemented, whereas the bootstrap-corrected SEs for Si and S were about 50% larger than their respective naïve estimates. The estimated biases resulting from the classical-like measurement error were so small as to be uninteresting from an epidemiologic perspective because the point estimates of all four pollutants after implementing measurement error correction were unchanged out to three decimal places.

**Table 5:** Point estimates ± SEs and 95% CIs for the different pollutants, using naïve analysis and with bootstrap correction for measurement error in covariate of interest.

PM_2.5_ component	Analysis/­correction	β̂_x_^*a*^±SE	95% CI
EC (μg/m^3^)	Naïve	0.001±0.014	–0.03, 0.03
	PB,^*b*^ λ=0	0.001±0.015	–0.03, 0.03
	PB, λ=1	0.001±0.015	–0.03, 0.03
OC (μg/m^3^)	Naïve	0.025±0.008	0.01, 0.04
	PB, λ=0	0.025±0.008	0.01, 0.04
	PB, λ=1	0.025±0.008	0.01, 0.04
Si (ng/m^3^)	Naïve	0.408±0.081	0.25, 0.57
	PB, λ=0	0.408±0.126	0.16, 0.66
	PB, λ=1	0.408±0.127	0.16, 0.66
S (μg/m^3^)	Naïve	0.055±0.017	0.022, 0.088
	PB, λ=0	0.055±0.025	0.006, 0.104
	PB, λ=1	0.055±0.025	0.006, 0.104
Point estimates are estimates of the increase in CIMT for a 1-unit increase in each pollutant. ^***a***^In the case of λ=1, β̂_x_ refers to the estimate corrected for any bias from ­classical-like error. ^*** b***^PB refers to results from parame­ter bootstrap implemented with given value of λ.			

## Discussion

*Summary.* Our comprehensive two-stage approach to estimating long-term effects of air pollution exposure includes a national prediction model to estimate exposures to individual PM_2.5_ components and corrects for measurement error in the epidemiologic analysis using a methodology that accounts for differing amounts of spatial structure in the exposure surfaces. In a case study of four components of PM_2.5_ and measurement error–corrected associations between these components and CIMT in the MESA cohort, corrected SEs corresponding to pollutants that exhibited significant spatial structure (i.e., Si and S) were 50% larger than naïve estimates, whereas corrected SE estimates for EC and OC were very similar to the naïve estimates.

*National exposure models.* We find that a national approach to exposure modeling is reasonable and performs well in terms of prediction accuracy. Our primary PLS + universal kriging models resulted in cross-validated *R*^2^ ≤ 0.95 (for predicting S concentrations) and ≥ 0.62 (for predicting Si) for any of the PM_2.5_ components. Use of kriging improved the cross-validated *R*^2^ for all four pollutants compared with models that used PLS only, although the improvement was not equal across all four pollutants. These results are useful in terms of understanding the spatial nature of our exposure surfaces. For EC and OC, the *R*^2^ only improved by ≤ 0.09 when kriging was used compared to when PLS alone was used, indicating little large-scale spatial structure in these pollutants. For Si, the *R*^2^ improved from 0.36 to 0.62; and for S, from 0.63 to 0.95. This indicates that S (and to a lesser extent Si) had substantial large-scale spatial structure that kriging was able to exploit. For all models, using kriging improved *R*^2^, indicating that no prediction accuracy was lost (and quite a bit stood to be gained, when spatial structure was present) by using PLS+universal kriging as opposed to using PLS alone. Our results also suggest that exposure models such as the ones we have built may be preferable in many cases to simpler approaches such as nearest-monitor interpolation. Our models produced cross-validated *R*^2^ that were higher than the nearest-monitor approach, and our results indicate that unless there is considerable spatial structure in the exposure surface, a substantial amount of prediction accuracy may be lost when the nearest-monitor approach is used.

We used two-stage modeling instead of joint modeling of exposure and health for a variety of reasons. One is pragmatic: Joint modeling is computationally intensive, so our two-stage approach is especially desirable when modeling multiple pollutants. Joint modeling may also be more sensitive to outliers in the health data. Two-stage modeling also appeals more intuitively in the context of modeling multiple health outcomes because it assigns one exposure per participant that can then be used to model a number of different health outcomes. Joint modeling, on the other hand, would assign different levels of the same pollutant depending on what health outcome was being modeled.

*Epidemiologic case study.* In this case study, we focused on four PM_2.5_ components selected to gain insight into the sources or features of PM_2.5_ that might contribute to the effects of PM_2.5_ on cardiovascular disease. EC and OC were chosen as markers of primary emissions from combustion processes, with OC also including contributions from secondary organic aerosols formed from atmospheric chemical reactions; Si was chosen as a marker of crustal dust; and S was chosen as a marker of sulfate, an inorganic aerosol formed secondarily from atmospheric chemical reactions (Vedal et al., in press). The mechanisms whereby exposures to PM_2.5_ or PM_2.5_ components produce cardiovascular effects such as atherosclerosis are not well understood, although several mechanisms have been proposed (Brook et al. 2010). [For discussion of other studies examining the effects of these pollutants, see Vedal et al. (in press).]

The relatively poor performance of nearest-monitor interpolation for EC, OC, and Si raises concerns about epidemiologic inferences based on predictions derived from that method. For S, the only pollutant for which our models and nearest-monitor interpolation performed comparably, the estimated increase in CIMT for a 1-unit increase in exposure based on nearest-monitor interpolation was 0.074 ± 0.018, comparable to the naïve inference made using predictions from our exposure models (0.055 ± 0.017). However, there is no way to correct for measurement error using this method, which is another significant advantage of our models.

Naïve health analyses based on exposure predictions from our national models indicated significant associations of CIMT with 1-unit increases in average OC, Si, and S, but not EC. Using the parameter bootstrap to account and correct for measurement error led to noticeably larger SEs and wider CIs for Si and S; however, OC, Si, and S were still significantly associated with CIMT even after correcting for measurement error.

*Measurement error correction.* For EC and OC, using PLS alone was sufficient to make accurate predictions, whereas the spatial smoothing from universal kriging substantially improved prediction accuracy for Si and S. It is accordingly no coincidence that the bootstrap-corrected SE estimates for EC and OC were unchanged from the naïve estimates, whereas the corrected SE estimates for Si and S were about 50% larger (and the resulting 95% CIs 50% wider) than their respective naïve estimates. The fact that the EC and OC exposure predictions were derived mostly from the PLS-only models, which assumed independent residuals, implies that the Berkson-like error was almost pure Berkson error (i.e., independent across location), which was correctly accounted for by naïve SE estimates. On the other hand, much more smoothing took place for Si and S, which induced spatial correlation in the residual difference between true and predicted exposure. Accordingly, SEs that correctly account for the Berkson-like error in these two pollutants are inflated because the correlated errors in the predictions translate into correlated residuals in the disease model that are not accounted for by naïve SE estimates (Szpiro et al. 2011b). The fact that the SE estimates from the parameter bootstrap using λ = 1 (which accounts for both Berkson-like and classical-like error) and using λ = 0 (which accounts only for Berkson-like error) were so similar further indicates that the larger corrected SE estimates were most likely a result of the Berkson-like error. None of our measurement error analyses indicated that any important bias was induced by the classical-like error.

*Limitations and model considerations.* Although our exposure models performed well, there is still room for improvement in prediction accuracy, especially for the EC, OC, and Si models, which had cross-validated *R*^2^ that could be improved upon. For these models it is possible that inclusion of additional geographic covariates in the PLS would help improve model performance. Examples include wood-burning sources within a given buffer for EC and OC concentrations, or dust and sand sources for Si. These covariates are currently not available in our databases. Furthermore, although it is possible to interpret the individual covariates in PLS components (Figure 2), such interpretations need to be regarded with caution because inclusion of many correlated covariates can lead to apparent associations that are counter-intuitive and the opposite of what might be expected scientifically. Finally, PLS does not consider interactions or nonlinear combinations of the geographic covariates, factors which could improve model performance.

*Implications and future directions.* Our results show that careful investigation of the exposure model characteristics can help to clarify the implications for the subsequent epidemiologic analyses that use the predicted exposures. As noted by Szpiro et al. (2011a), an overarching framework that considers the end goal of health modeling seems more appealing than treating exposure models as if they exist for their own sake. This analysis serves as an example that will inform ongoing efforts by our group and others to construct and utilize exposure prediction models that are most suitable for epidemiologic studies.

Our epidemiologic inference was based on one health model per pollutant. One might reasonably be interested in how multiple pollutants jointly affect health. However, current literature for measurement error correction does not address models that use multiple predicted pollutants as exposures. Our group is currently working on methods to address this challenge.

## Supplemental Material

(2.4 MB) PDF
