# Supplementary material for: A National Prediction Model for PM2.5 Component Exposures and Measurement Error–Corrected Health Effect Inference
Source: Environ Health Perspect. 2013 Jun 11;121(9):1017–25. doi: 10.1289/ehp.1206010 (PMC3764074; doi:10.1289/ehp.1206010)
Supplement: (2.4 MB) PDF [file ehp.1206010.s001.pdf]

*Environmental Health Perspectives* doi:10.1289/ehp.1206010

# Supplemental Material

# A National Prediction Model for $PM_{2.5}$ Component Exposures and Measurement Error–Corrected Health Effect Inference

Silas Bergen, Lianne Sheppard, Paul D. Sampson, Sun-Young Kim, Mark Richards, Sverre Vedal, Joel D. Kaufman, and Adam A. Szpiro

## Table of Contents

Implementation of simulation extrapolation ........ 3

Supplemental Material, Figure S1 ........ 5

References ........ 6

**Implementation of simulation extrapolation**

The extension of the parameter bootstrap discussed in Methods under *Measurement Error Correction* wherein we sample $(\hat{\boldsymbol{\alpha}}, \log(\hat{\boldsymbol{\theta}}))$ from a covariance multiplied by a non-negative λ provides additional insight into how to estimate the bias of resulting from the classical-like error, and can be thought of in the framework of simulation extrapolation (SIMEX) (Stefanski et al. 1995). Consider first the parameter bootstrap with λ=0. Though we use the originally estimated parameters $(\hat{\boldsymbol{\alpha}}, \log(\hat{\boldsymbol{\theta}}))$ throughout, these original estimates are a realization from a sampling distribution with some amount of variance, which can be thought of heuristically as "one unit of variance." The bias estimate from the parameter bootstrap with λ=1 (corresponding to "two units of variance") assumes that the bias from the classical-like error obtained by going from λ=0 to λ=1 is the same as the bias induced by using the originally estimated parameters instead of the true, unknown parameters; in other words, the bias is treated as linear in λ. However if we perform the parameter bootstrap using different values of λ and estimate the bias for each one, we can get a more flexible representation of how the bias varies as a function of λ. Plotting realized $\widehat{Bias_{\lambda}}(\widehat{\beta_X})$ versus λ for several values of λ and extrapolating to $\widehat{Bias_{-1}}(\widehat{\beta_X})$ gives an alternative estimate of the bias. This extension is equivalent to performing a SIMEX analysis to extrapolate to the hypothetical setting where the variance of the measurement error is zero (Stefanski et al. 1995). We performed the parameter bootstrap using sample sizes of 30,000, sampling $(\hat{\boldsymbol{\alpha}}_j, \log(\hat{\boldsymbol{\theta}}_j))$ from the inverse Hessian inflated by factors of λ∈{0,0.5,1,1.5,2} and plotted the corresponding $\widehat{Bias_{\lambda}}(\widehat{\beta_X})$ against these values of λ. We then performed both linear and quadratic extrapolation to $\widehat{Bias_{-1}}(\widehat{\beta_X})$. The SIMEX-corrected estimate of $\widehat{\beta_X}$ is defined as:

$$\widehat{\beta_{X,S}} = \widehat{\beta}_X + \widehat{Bias_{-1}}(\widehat{\beta_X})$$

This estimate was compared to the other corrected estimates defined in Methods under *Measurement Error Correction*.

Figure S1 shows the results of the SIMEX implementation of the parameter bootstrap using linear and quadratic extrapolation. We see that the choice of extrapolating function slightly affected the SIMEX estimate of the bias for all four of the pollutants, though the bias was so small that the differences between the extrapolating function were trivial. Overall, while the SIMEX bias corrections did not suggest any meaningful bias for any of the pollutants, all of these plots suggest that the bias from classical-like measurement error is away from the null, similar to previously published simulation results (Szpiro et al. 2011). This is different from the usual bias toward the null from classical measurement error, confirming that additional caution is needed in the air pollution setting since we cannot always assume that ignoring measurement error results in conservative inference.

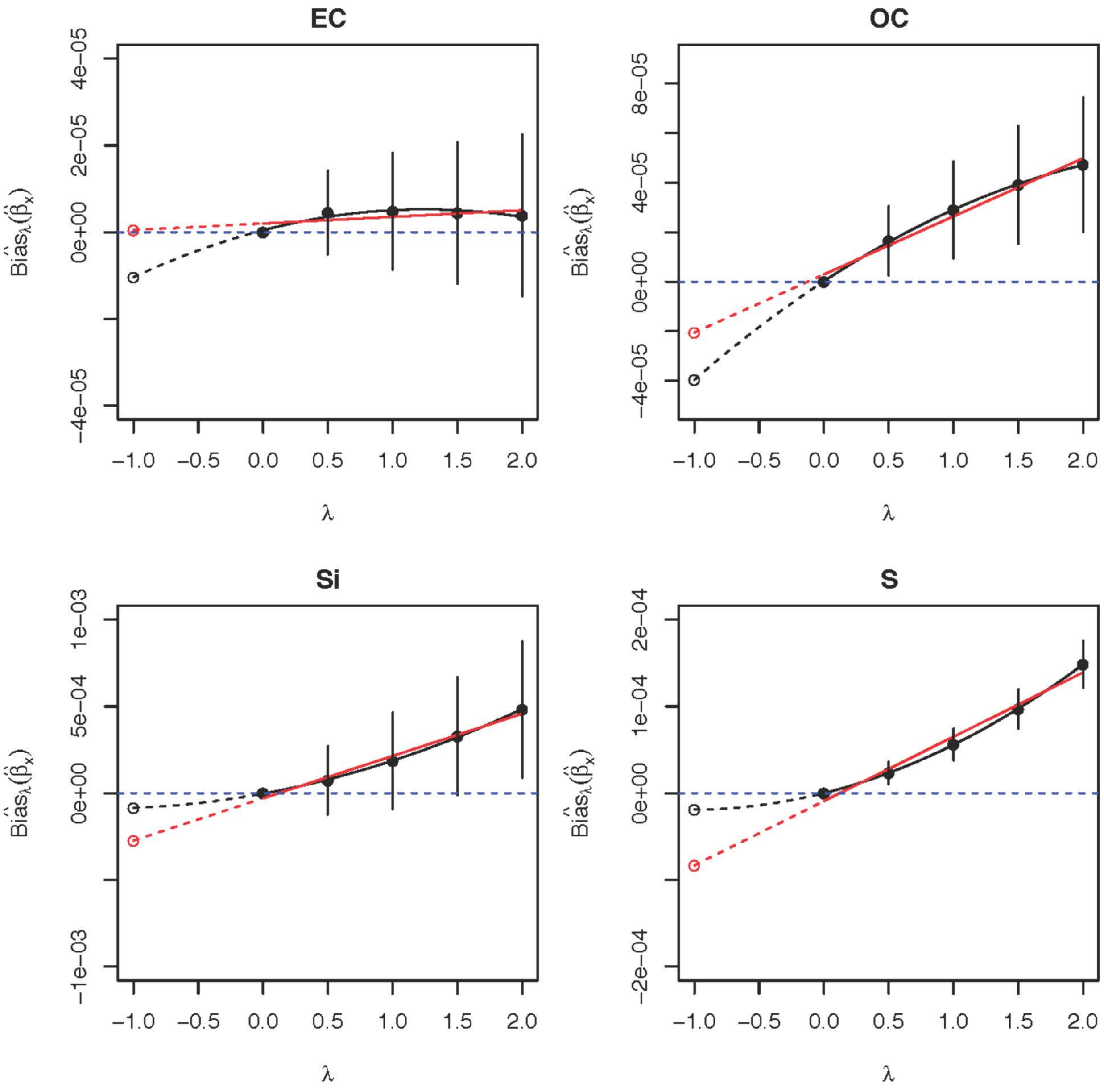


**Supplemental Material, Figure S1:** SIMEX bias estimates. 30,000 parameter bootstrap samples were drawn using values of $\lambda \in \{0,0.5,1,1.5,2\}$, and the estimated bootstrap biases plotted as a function of these values. A linear or quadratic extrapolation was used to estimate $\widehat{Bias_{-1}}(\widehat{\beta_X})$. Confidence intervals from a t-test testing zero bias are also shown. The four panels correspond to components as follows: (a) EC, (b) OC, (c) Si, and (d) S.

## References


Stefanski, LA and Cook, JR. 1995. Simulation-extrapolation: the measurement error jackknife. J Am Stat Assoc, 90(432):1247–1256.

Szpiro AA, Sheppard L, and Lumley T. 2011. Efficient measurement error correction with spatially misaligned data. Biostatistics, 12(4):610–623.
